# Special Issue “Molecular Immunology of the Male Reproductive System”

**DOI:** 10.3390/ijms25041981

**Published:** 2024-02-06

**Authors:** Gerhard Haidl

**Affiliations:** Department of Andrology, University Hospital Bonn, Campus-Venusberg 1, 53127 Bonn, Germany; gerhard_haidl@t-online.de

The immunological aspects of male infertility have gradually become the focus of both basic and clinical research. Although inflammatory and immunological effects on the male genital tract are increasingly acknowledged as significant etiologic factors involved in male infertility, they are still unsatisfactorily recognized in the diagnostic workup of infertile couples [1]. One reason is a considerable gap between basic research and its translation into clinical practice.

The articles in this Special Issue all address basic research but concomitantly investigate the associations with the clinically relevant aspects of the human male reproductive system, including approaches for better management. All organs of the male genital tract–from the testis to the prostate—are addressed. In one contribution, the role of apoptosis in chronic asymptomatic orchitis in dogs is explored, showing many parallels with the human system and suggesting treatment options through the prevention of apoptosis and the subsequent DNA fragmentation of sperm [2]. An important argument for the reduction in reactive oxygen species and the involvement of proinflammatory factors is made in a contribution demonstrating the influence of the C-natriuretic protein on sperm motility [3]. The widely overlooked clinical diagnosis of chronic epididymitis, due to its mainly asymptomatic course, is considered in another contribution highlighting the significance of the myeloid differentiation response gene 88 for inducing the inflammatory responses of dendritic cells in the epididymis, which has also a high impact on clinical treatment strategies [4]. Benign prostatic hyperplasia is also a major issue both in andrology and urology. New therapeutic targets may be evaluated in terms of androgen-independent autoimmunolgic reactions that can trigger BPH [5]. Lastly, in an article on the role of mononuclear phagocytes, the authors highlight their function as a control point for the maintenance of the immune tolerance of the testis and epididymis, as well as for the elimination of pathogens, which again is of high clinical relevance [6].

With five notable research contributions, this Special Issue aims to narrow the gap between basic research and the clinical implications of the immunological factors involved in male fertility.

Inflammatory and immunological factors are also observed in female fertility problems, for example, endometritis, which is associated with impaired implantation and pregnancy rates and an increased chance of miscarriage [7]. Thus, it cannot be excluded, that the signs of inflammation such as an increased number of natural killer cells in the endometrium are also associated with inflammatory processes in the male genital tract.

These considerations suggest the urgent need for more research efforts focused on immunological processes in both the female and male genital tracts, their potential interaction, and their impact on fertility and pregnancy.

This will hopefully be a topic of a future Special Issue.
